# Targeted Gene Deletion of miRNAs in Mice by TALEN System

**DOI:** 10.1371/journal.pone.0076004

**Published:** 2013-10-16

**Authors:** Shuji Takada, Tempei Sato, Yoshiaki Ito, Satoshi Yamashita, Tomoko Kato, Miyuri Kawasumi, Masami Kanai-Azuma, Arisa Igarashi, Tomomi Kato, Moe Tamano, Hiroshi Asahara

**Affiliations:** 1 Department of Systems BioMedicine, National Research Institute for Child Health and Development, Tokyo, Japan; 2 Department of Systems BioMedicine, Graduate School of Medical and Dental Sciences, Tokyo Medical and Dental University, Tokyo, Japan; 3 Center for Experimental Animal, Tokyo Medical and Dental University, Tokyo, Japan; 4 CREST, Japan Science and Technology Agency (JST), Saitama, Japan; 5 Department of Molecular and Experimental Medicine, The Scripps Research Institute, La Jolla, California, United States of America; Academia Sinica, Taiwan

## Abstract

Mice are among the most valuable model animal species with an enormous amount of heritage in genetic modification studies. However, targeting genes in mice is sometimes difficult, especially for small genes, such as microRNAs (miRNAs) and targeting genes in repeat sequences. Here we optimized the application of TALEN system for mice and successfully obtained gene targeting technique in mice for intergenic region and series of microRNAs. Microinjection of synthesized RNA of TALEN targeting each gene in one cell stage of embryo was carried out and injected oocytes were transferred into pseudopregnant ICR female mice, producing a high success rate of the targeted deletion of miRNA genes. In our condition, TALEN RNA without poly(A) tail worked better than that of with poly(A) tail. This mutated allele in miRNA was transmitted to the next generation, suggesting the successful germ line transmission of this targeting method. Consistent with our notion of miRNAs maturation mechanism, in homozygous mutant mice of miR-10a, the non- mutated strand of miRNAs expression was completely diminished. This method will lead us to expand and accelerate our genetic research using mice in a high throughput way.

## Introduction

To explore the region-specific genome functions *in vivo*, generation of gene-targeting animals is a powerful strategy. In this regard, enormous amount of endeavors was used for generating knockout mice, since embryonic stem cells (ES cells) for homologous recombination or nuclear transfer technologies are established firstly in mice species. It should be also noted that compared with other model animals, mice have several strong advantages, such as in the size of body and the fertilization cycle, genetically close relationship to human genome, exist of inbred strains. In fact, as post genome study, worldwide consortiums are trying to generate almost all the genes targeting ES lines [1]. However, recent drastic progress of molecular biology, including non-coding RNA discovery and spatial-temporal enhancer research, ask us for creating even further amount of and complicated genome deletion mice, including large scale generation of microRNA (miRNA) targeting mice [2], which are sometimes difficult to generate with conventional methods. Recently developed Zinc-finger nuclease (ZFN) and transcription activator-like effector nuclease (TALEN) are powerful tools to generate knockout animals in several model species including zebrafish, *Xenopus* and rats [3]-[9]. One of the merits of this system is that they are free from using ES cells. The genome of fertilized zygotes was directly modified by injection of artificial endonuclease, such as ZFNs and TALENs. ZFNs and TALENs are fusion proteins containing DNA-binding domain and FokI endonuclease at the C-termini. The recognition sequences of DNA-binding domain can be designed with some restrictions such as ZFNs require (GNN)_n_ sequences and TALENs T at 3′ end of the recognition sequences. When two ZFNs or two TALENs bind to their target sequences in a genome in an appropriate orientation and distance, FokI part of the proteins are dimerized and cause double-strand break, resulting in stimulation of the breakage repair mechanisms in cell nuclei [10]–[12]. The breakage is repaired by non-homologous end-joining (NHEJ), which frequently results in small insertions or deletions [13], [14].

Although ZFNs and TALENs are basically compatible for application, TALENs have some advantages over ZNFs; TALENs are more flexible for designing and assembling recognition sequences [15], cost and time are efficient than ZNFs and construction for TALENs is freely available from Addgene (http://www.addgene.org). Actually, TALENs have been applied for making knockout mice for several genes [16]–[18]. Also some genes for miRNAs have been targeted in human cell line using TALENs [19].

Taken together, application of TALENs to mice genetic studies are urged and in this regard, here we report optimized high-efficient genome targeting protocol in mice by TALEN system.

## Materials and Methods

### Evaluation of TALENs activities in cultured cell line

We used custom TALEN Access service (Cellectis bioresearch) to design and construct plasmids coding for TALEN with an option of CMV and T7 vector. Target sequences of TALENs are as follows; TGGGGCCTCCAGGAGCC and TGTTGATTTTGTGGTTT for the gene desert on mouse chromosome 11, TCTGTGTGTATCCCCAG and TTGATATAACCCATGGA for *mmu-mir-146a*, TCTGTATATACCCTGTA and TGACCACAAAATTCCTT for *mmu-mir-10a* and TGTAACGTTGTCTATAT and TGGGTACCACACAAATT for *mmu-mir-10b*. Each pair of TALEN plasmids and pIRES2-EGFP were co-transfected to NIH3T3 cell line (ATCC) (2×10^4^) using 24-well plate. After 2 days of culture, G418 at final concentration of 500 µg/ml was added to each well. G418 resistant cells were harvested and lysed using 10 mM Tris-HCl (pH 7.5)/1 mM EDTA (pH 8.0)/1% SDS/100 µg/ml Proteinase K. Samples were incubated at 50°C for 30 min, extracted by phenol/chloroform and diluted using distilled water. PCR was performed using primers as follows: coF (5′-GCG GAT CCT GCA GAG AGA TGA ATA ACA A-3′) and coR (5′-GCA GAT CTT TGA TAT TTC ACA AAG TAG-3′) for the gene desert on mouse chromosome 11, miR-146a-F (5′-CCA CTC TTC AAG TTC CTT GGC AG-3′) and miR-146a-R (5′-GAT TGC TTA TGA ACT TGC CTA TCT TGT G-3′) for *mmu-mir-146a*, 10aF (5′-TTG CAC AAC AGC TGC CTT T-3′) and 10aR (5′-AAC AAG GAC CCA AGC TTC CA-3′) for *mmu-mir-10a* and 10bF (5′-AAG AAG GTC CTG GCT GCT CA-3′) and 10bR (5′-TCT CCA GGA AAA GGC TGT ACA AT-3′) for *mmu-mir-10b*. PCR products obtained were cloned into pCR4-TOPO vector using TOPO TA Cloning Kits for Sequencing (Invitrogen). Inserts of plasmids were PCR amplified using M13 forward and M13 reverse primers. Sequences were identified using BigDye terminator version 3.1 (Life Technologies) and ABI3500 Genetic Analyzer (Life Technologies) after ExoSAP-IT (Affymetrix) treatment. Primers used were coR for the gene desert on mouse chromosome 11 and M13 reverse for *mmu-mir-146a*, *mmu-mir-10a*, and *mmu-mir-10b*.

### Microinjection

TALEN plasmids were linearized by PacI endonuclease digestion. One microgram of linearized plasmid was used as a template for in vitro transcription reaction using mMESSAGE mMACHINE T7 Kit (Life Technologies) according to the manufactures instruction. To add poly(A) tail to the RNA, synthesized RNA was further treated with Poly(A) Tailing Kit (Life Technologies) according to the manufactures instruction. The RNA with or without poly(A) was purified by MegaClear kit (Life Technologies) according to the manufactures instruction. The RNA concentration was determined by absorption and diluted with injection buffer (10 mM Tris-HCl/0.1 mM EDTA (pH 7.4)) at desired concentration. Microinjection of a mixture of RNAs into cytoplasm of one cell stage embryo was carried out under standard procedures using oocytes obtained from superovulated (C57BL/6 x DBA2) F1 mice (Sankyo Labo Service Corporation). Injected oocytes were cultured in M16 medium. Next day embryos developed to two-cell stage were transferred into pseudopregnant ICR female mice. The protocols for animal experiments were approved by the Animal Care and Use Committee at National Research Institute for Child Health and Development (Permit Numbers: A2004-003-C09, A2009-002-C04), Tokyo Medical and Dental University (Permit Number: 0130160A, 0140086A) and The Scripps Research Institute (Permit Number: 09-0029).

### Genotyping

Genomic DNA was extracted from york sac of E13.5 embryos or tail tips of pups. PCR was performed as above. PCR products obtained were treated with ExoSAP-IT and used as templates for sequencing and T7 endonuclease I (T7EI) assay. Sequencing primers used were coR for the gene desert on mouse chromosome 11, miR-146a-R for *mmu-mir-146a*, 10aF for *mmu-mir-10a* and 10bF for *mmu-mir-10b*. To confirm mutant alleles in nucleotide levels, independent PCR and direct sequencing was carried out and the PCR product was cloned and sequenced as above. For T7EI assay, denatured and re-annealed PCR products were digested with T7EI and then analyzed by agarose gel electrophoresis. Band intensities were quantified by MultiGauge version 3.2 software (Fujifilm Corporation).

### Quantitative real-time RT-PCR

Total RNA was purified from kidneys of 4-week-old mice using ISOGEN (Nippongene) according to the manufactures instruction. Reverse transcription (RT) and PCR were carried out using TaqMan MicroRNA RT Kit (Life Technologies), TaqMan Universal PCR Master Mix II, w/o UNG (Life Technologies) and TaqMan micoRNA assay (Life Technologies). As an internal control TaqMan against U6 (RNU6B) was used. ▵▵Ct method was adopted for calculation of relative gene expression levels. Expression levels were obtained from average of duplicate trial.

## Results

### Evaluation of TALENs activities in cultured cell line

The aim of this study is generation of knockout mouse deficient for a gene desert and miRNAs using TALEN through microinjection of fertilized oocytes. As a first step, we examined if TALEN technology is useful for introduction of mutation on a gene desert and miRNA loci on mouse genome. To test this, we ordered construction of TALENs for specific gene desert of mouse genome, which is located on chromosome 11, *mmu-mir-146a*, *mmu-mir-10a* and *mmu-mir-10b* from Cellectis bioresearch. The activity of the TALENs were evaluated by the company using yeast single strand annealing (SSA) assay, which is a method for measuring genome cleavage activity ranged 0-1, where high number indicates high activity and value of 0.45 or higher means good cutter. The value of SSA assay for TALEN pairs we obtained were 0.86 for the gene desert, 0.64 for *mmu-mir-146a*, 0.92 for *mmu-mir-10a* and 0.75 for *mmu-mir-10b*, showing that all TALEN have cleavage activity in yeast.

**Figure 1:**
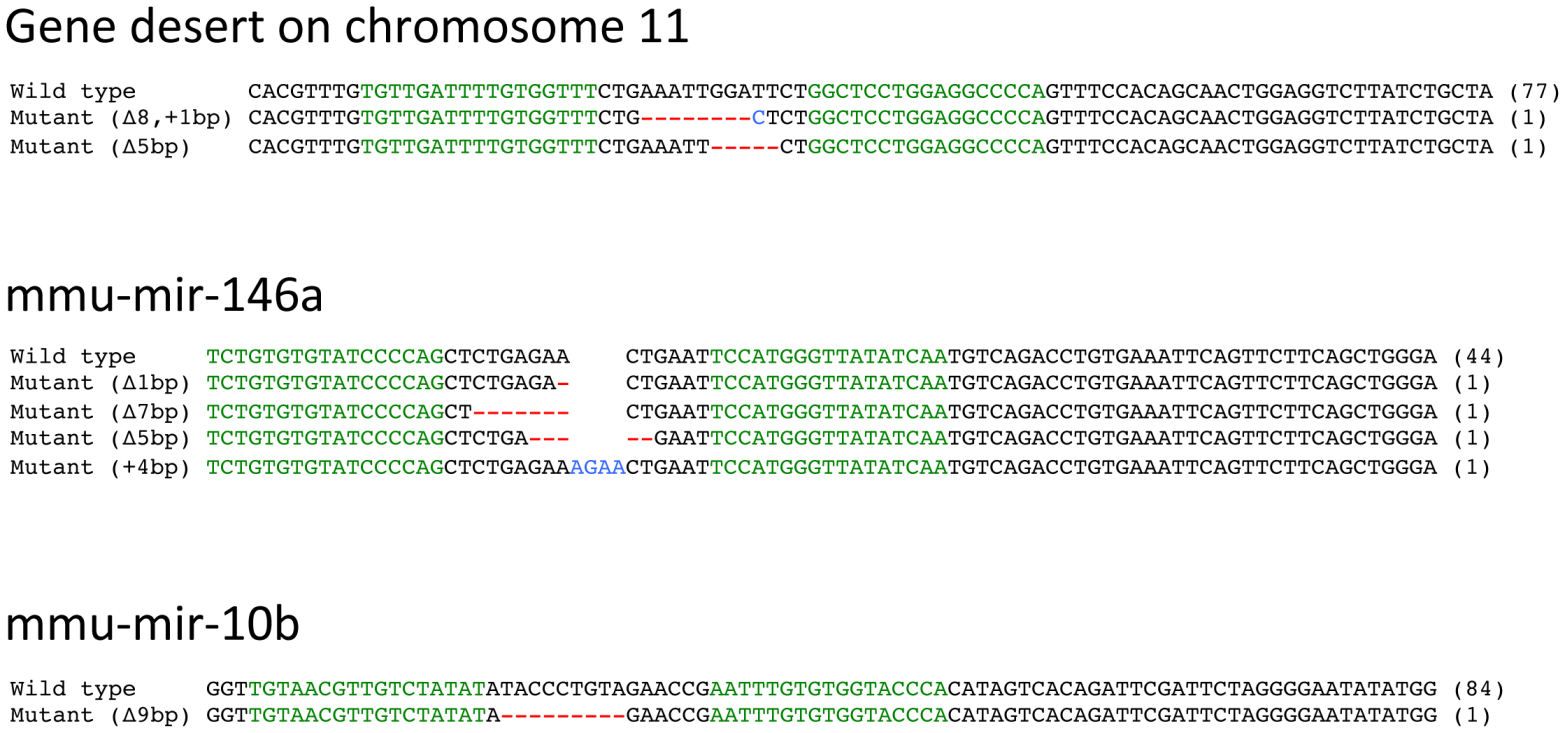
Cell based assay of TALEN activities. Each sequence shows the result of PCR direct sequencing of cloned PCR products amplified from TALEN transfected cells. Green letters designate target sequences of TALENs. Red dashes and blue letters indicate deleted and inserted nucleotides, respectively. The sizes of deletions are shown to the right of mutated alleles with ▵ and/or +. Alleles without mutation are indicated with Wild type. The numbers in the parenthesis indicate the number of clones obtained.

We examined the genome editing activities of TALEN pairs for the gene desert, *mmu-mir-146a*, *mmu-mir-10a* and *mmu-mir-10b* in NIH3T3 cultured cell line. Since the TALEN plasmids does not contain drug resistant gene for mammalian cell line, pIRES2-EGFP, which contains neomycine resistant gene, was co-transfected with TALEN plasmids to NIH3T3 to increase TALEN introduced cell populations by G418 selection. To evaluate efficiencies of genome editing, nucleotide sequences of TALEN cleavage sites were identified by PCR amplification followed by cloning of PCR products from TALEN transfected cells. Sequencing analysis showed that the genome editing activities were confirmed at rate of 2.5% (2 mutated sequences out of 79 sequences) for the gene desert on chromosome 11, 8.2% (4 mutated sequences out of 48 sequences) for *mmu-mir-146a* and 1.2% (1 mutated sequence out of 85 sequences) for *mmu-mir-10b* while no mutated sequence was identified from 207 sequences for *mmu-mir-10a* (Fig. 1), indicating that the results are not always parallel between this method and yeast SSA assay in that TALEN pair for *mmu-mir-146a* shows the highest activity in former assay whereas lowest in latter among four TALEN pairs. This result implying that at least three TALEN pairs, for the gene desert, *mmu-mir-146a* and *mmu-mir-10b*, could be useful for making knockout mouse.

### Optimization of TALEN mRNA injection method

**Figure 2:**
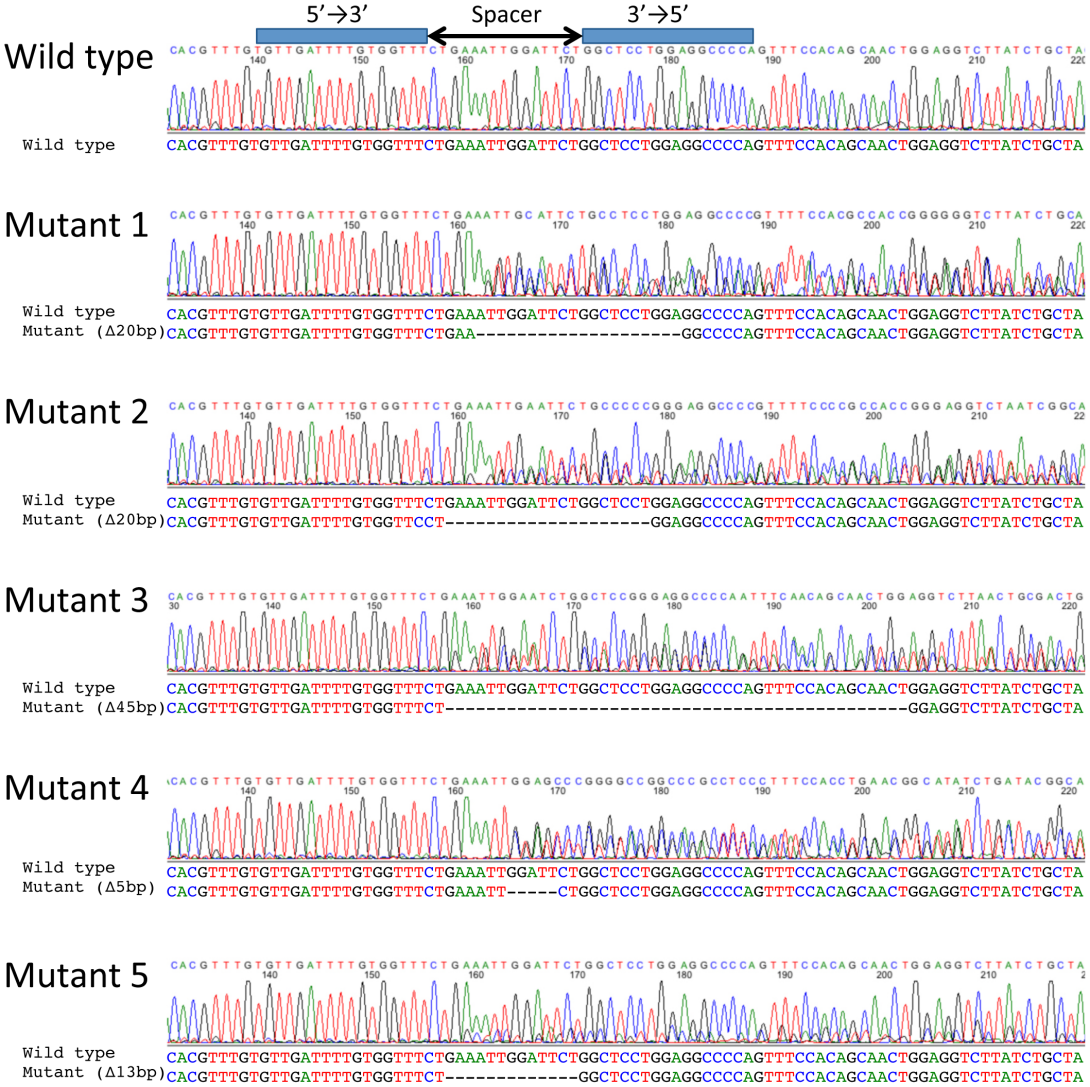
PCR genotyping of the gene desert on chromosome 11 mutants. Genomic sequence is shown at the top with the target sequences of a pair of TALEN colored in green. Nucleotide sequences of mutated alleles are indicated with red dashes. The sizes of deletions are shown to the right of mutated alleles with ▵. Alleles without mutation are indicated with Wild type.

To make knockout mouse using TALEN technology, we optimized TALEN mRNA microinjection method in mouse fertilized oocytes using TALEN pairs for the gene desert with different conditions; RNA synthesized with or without poly (A) tail; concentration of RNA for injection into cytoplasm of pronuclear-stage oocytes. We injected TALEN mRNAs without poly (A) tail at 10 ng/µl to 190 oocytes. One hundred sixty nine embryos entered to 2-cell stage the next day and transferred into oviduct of pseudo-pregnant female (Table 1). Embryos were collected at E13.5 and genotypes were determined using DNAs prepared from tails with PCR direct sequencing. As a result, no mutant was obtained. It may be possible that RNA concentration was not enough for inducing mutation using TALEN RNAs, therefore 50 and 100 ng/µl of TALEN RNAs without poly (A) tail were used for microinjection. 38 and 40 oocytes were injected, 22 and 23 embryos entered to 2-cell stage and 17 and 20 of E13.5 embryos were obtained using 50 and 100 ng/µl of TALEN RNAs, respectively (Table 1). Genotyping showed that no mutant was produced. We next tried to use several concentrations of TALEN mRNAs with poly (A) tail. TALEN RNAs at the concentration of 10, 50 and 100 ng/µl were injected to 52, 58 and 58 oocytes, and 41, 20 and 40 embryos developed to 2-cell stage and 26, 16 and 30 of E13.5 embryos were obtained, respectively (Table 1). Genotyping showed that no mutant was produced. It may be that TALEN RNAs at the concentration of 100 ng/µl is not still sufficient for producing mutant mice or the number of microinjection is too small to obtain a mutant because the efficiency of TALEN RNAs at that concentration is too low for inducing mutation. Finally we injected TALEN mRNAs with or without poly (A) tail at the concentration of 100 ng/µl. Microinjection was carried out using 155 and 196 oocytes, 82 and 138 embryos developed 2-cell stage and 56 and 73 embryos were collected at E13.5 (Table 1). Genotyping showed that no mutant was obtained from TALEN mRNAs with poly (A) tail injected embryos whereas 5 embryos with different mutations (out of 56 embryos; success rate is 8.9%) were identified from TALEN mRNAs without poly (A) tail injection (Fig. 2). Mutations were further re-confirmed by sequencing after independent round of PCR. From this result TALEN mRNAs without poly (A) tail seems to work better for inducing mutation than TALEN mRNAs with poly (A) tail. We used TALEN mRNAs without poly (A) tail at concentration of the 100 ng/µl was used for following experiment.

**Table 1:** Gene targeting efficiencies.

TALEN	With or without poly(A) tail	Dose of TALEN mRNA (ng/µl)	Number of injected oocytes	Number of Two-cell embryos	Number of embryos or new born obtained	Number of mutants
**Gene desert on chromosome11**	without	10	190	167	75	0
**Gene desert on chromosome11**	without	50	38	22	17	0
**Gene desert on chromosome11**	without	100	40	23	20	0
**Gene desert on chromosome11**	with	10	52	41	26	0
**Gene desert on chromosome11**	with	50	58	20	16	0
**Gene desert on chromosome11**	with	100	58	40	30	0
**Gene desert on chromosome11**	without	100	155	82	56	5
**Gene desert on chromosome11**	with	100	196	138	73	0
**mmu-mir-146a**	without	100	63	44	4	2
**mmu-mir-146a**	without	100	52	42	8	5
**mmu-mir-146a**	without	100	30	25	3	0
**mmu-mir-146a**	without	100	65	56	12	1
**mmu-mir-10a, mmu-mir-10b** *	without	100, 100	62	42	15	0, 0†
**mmu-mir-10a, mmu-mir-10b** *	without	200, 200	69	60	19	0, 0†
**mmu-mir-10a, mmu-mir-10b** *	without	400, 400	131	105	48	0, 2†
**mmu-mir-10a**	without	500	59	32	5	0
**mmu-mir-10a**	without	500	181	96	54	3
**mmu-mir-10a**	without	500	174	120	28	1

* TALENs for *mmu-mir-10a* and *mmu-mir-10b* were mixed and microinjected. †Genotype of *mmu-mir-10a* and *mmu-mir-10b* were assayed and results were indicated separately in a cell.

### Production of mmu-mir-146a knockout mouse

**Figure 3:**
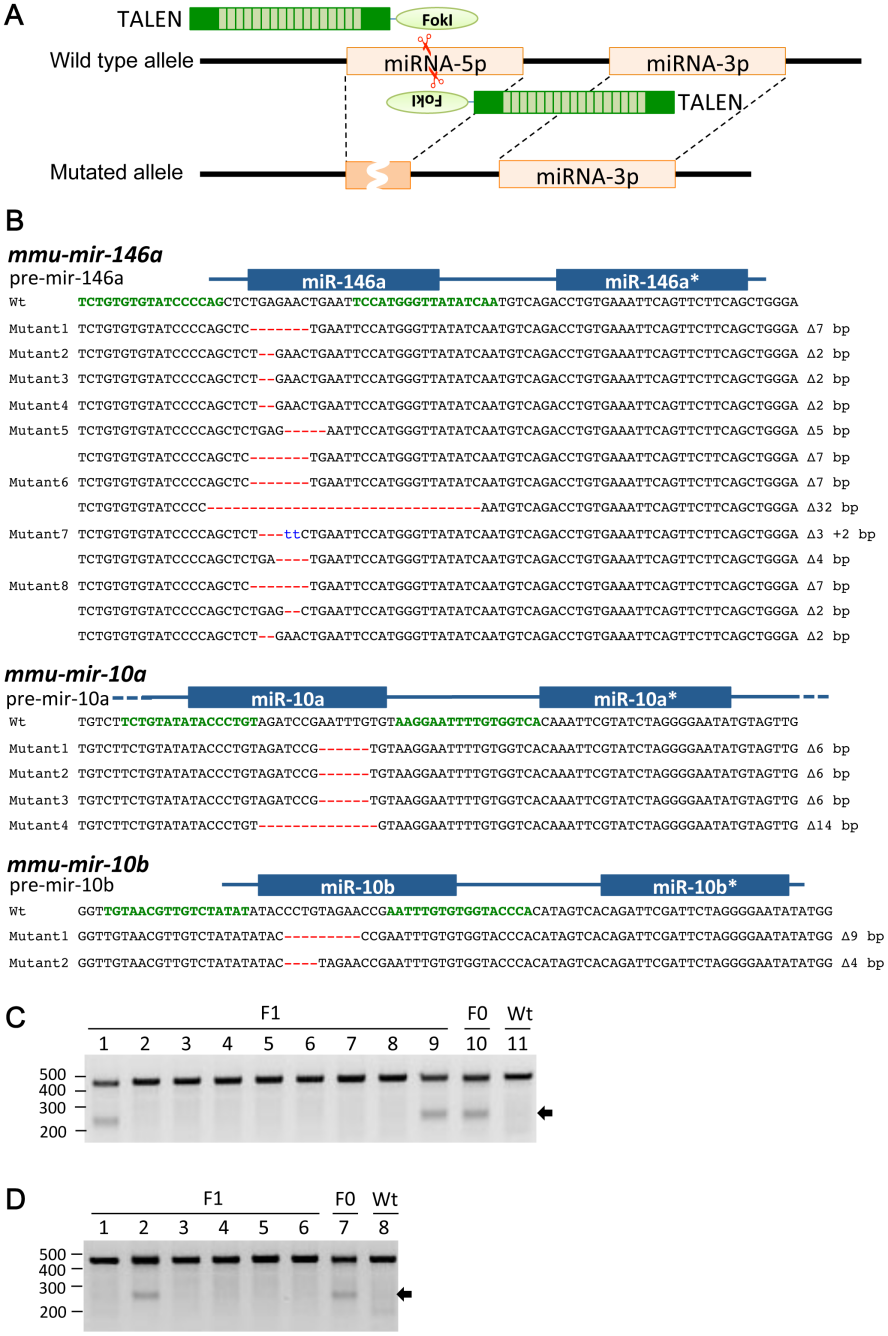
Production of microRNA deficient mice with TALEN. (A) Schematic representation of TALENs that target microRNA. Wild type genomic sequence is shown with black line. miRNA-5p and -3p on the genomic sequence are with orange boxes. TALENs are indicated with green boxes and recognition sequences are indicated with light green boxes. Scissors show the location of cutting site of FokI endonuclease. Genomic sequence of mutated allele is indicated with black line at the bottom. Deleted sequence is shown with a dark orange box with a white waved line. (B) PCR genotyping of the miRNA mutants. Genomic sequence is shown at the top with the target sequences of a pair of TALEN colored in green. Blue lines and boxes indicate pre-miRNAs and mature-miRNAs sequences, respectively. Deleted and inserted nucleotides are indicated with red dashes and blue letters, respectively. The sizes of deletions and insertions are shown to the right of mutated alleles with ▵ and +, respectively. Alleles without mutation are indicated with wt (wild type). (C) Genotyping of F1 offspring of *mmu-mir-10a* KO (Mutant3) mouse by T7EI assay. Arrows indicate the digested PCR products containing mutation. DNA size marker is shown at left. Lane 1-9: F1 individuals; lane 10: founder; lane 11: Wt. (D) Genotyping of F1 offspring of *mmu-mir-10b* KO (Mutant1) mouse by T7EI assay. Arrows indicate the digested PCR products containing mutation. DNA size marker is shown at left. Lane 1-6: F1 individuals; lane 7: founder; lane 8: Wt.

Utilizing above condition, we set out to make a knockout mouse deficient in series of miRNAs; *mmu-mir-146a*, *mmu-mir-10a,* and *mmu-mir -10b.* Genotyping of pups derived from the injected oocytes by PCR direct sequencing showed 8 out of 27 individuals (29.6%) contain wild type and mutated alleles in miR-146a (no biallelically modified mice were obtained so far) (Fig. 3B). One type of mutated allele was identified in four pups respectively (Mutant1-4, Fig. 3B), whereas two types of mutated alleles were identified in three pups respectively (Mutant5-7, Fig. 3B) and four were identified in one pup (Mutant8, Fig. 3B), implying that the TALEN protein worked at one-cell stage embryos in the former four cases and at two-cell stage or after in the latter four cases.

### Production of mmu-mir-10a and -10b knockout mice

We next tried to generate *mmu-mir-10a* and *-10b* deficient mice. To this end, mRNAs without polyA synthesized from TALEN plasmids for *mmu-mir-10a* and *-10b* were mixed and injected into fertilized oocytes at the concentration of 100, 200 and 400 ng/µl each for single TALEN pair. Genotyping of obtained pups showed that there was not a mutant mouse by microinjection at the concentration of 100 and 200 ng/µl each for single TALEN pair, whereas two mutant mice were obtained from injection at 400 µg/ml (Table 1). Both mutants have mutated *mmu-mir-10b* and wild type *mmu-mir-10a* alleles (Fig. 2B). Higher concentration of RNA mixture for microinjection enabled us to produce *mmu-mir-10b*, prompted us to try microinjection of TALEN mRNAs at the concentration of 500 ng/µl for generating *mmu-mir-10a* deficient mice. Genotyping of the pups thus obtained showed that 3 out of 87 (3.4%) in total contained mutated alleles (Table 1, Fig. 3B), suggesting that TALEN activity is better in fertilized oocyte than NIH3T3 because we could not identify any mutated sequence by TALEN transfected NIH3T3 genome. Taken together, the optimum concentrations of RNA derived from TALEN plasmids could be different from design to design, probably because efficiencies of TALENs are different among target sequences of TALENs.

### Transmission of the mutated allele to the next generation

To examine if the mutated alleles induced by microinjection of TALEN RNAs can be transmitted to next generation or not, two independent heterozygous mice deficient for *mmu-mir-10a* and *-10b* were mated with wild type C57BL/6 strain. Genotyping of the F1 pups was performed using T7EI assay. As shown in Fig. 3C and 3D, mutated *mmu-mir-10a* and *-10b* allele were identified. Ratios of the intensities of digested/undigested bands were quantified, showing that lane 1, 9 and 10 of Figure 3C (*mmu-mir-10a* mutant 3) are similar (0.39, 0.39 and 0.37, respectively), and lane 2 and 7 in Figure 3D are similar (0.19 and 0.24, respectively). Since F1 pups should be heterozygous, it seems likely that founder of *mmu-mir-10a* mutant 3 and *mmu-mir-10b* mutant 1 are heterozygous mutants without mosaicism. This results indicate that mutations induced by TALENs are transmittable to next generations, suggesting that TALEN mediated mutagenesis of mouse genome can be useful for making homozygous knockout mice by mating F1 heterozygous mice.

### Expression levels

**Figure 4:**
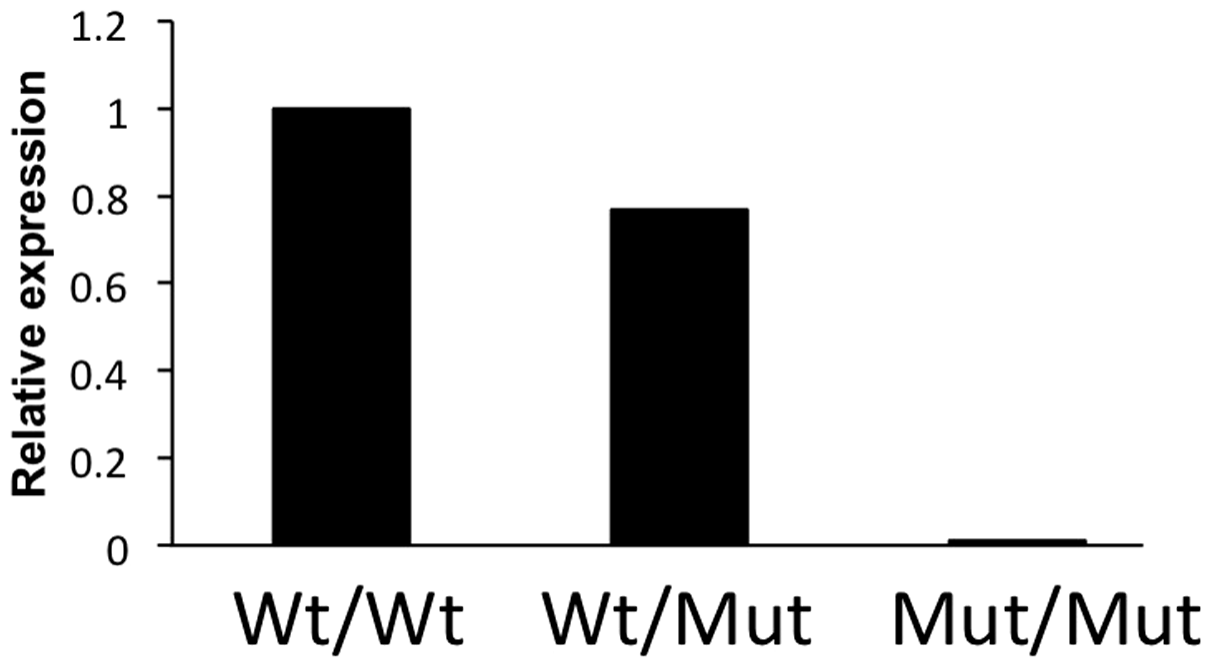
Real-time RT-PCR analysis of miR-10a*. Genotype of each animal is indicated at bottom. Mut indicates mutant allele. Expression level of the wt animal was adjusted as 1.

TALENs for miRNA knockout we used cleaved one of mature-miRNA sequence on miRNA precursor. It should be reasonable that mutated mature-miRNA itself, if not all alleles, does not function because several bases were deleted from short sequence of mature-miRNA. Also mutation on mature-miRNA may disturb precursor-miRNA to be processed to mature-miRNA because precursor-miRNA could not form hairpin structure. In this case remaining intact mature-miRNA located the other side of mutated mature-miRNA on the precursor molecule can not be expressed. We tested this possibility by RT-PCR analysis of mir-10a* using 6-base deleted mir-10a mutant mouse. As shown in Fig. 4, the expression level was reduced in wt/mutant hetero mouse and deleted in mutant homo mouse, suggesting that mutations introduced by TALEN at one of mature-miRNA is enough for deplete function for both mature-miRNAs on a precursor.

## Discussion

Our results demonstrate that TALENs can be applicable for generating knockout mice for gene desert and miRNA loci without using ES cells in high throughput way and in a short period of time, by optimizing RNA concentration for microinjection for each set of TALEN design. Also we showed that functions for both mature-miRNAs on a precursor can be depleted by introduction of mutations by TALEN at one of mature-miRNA.

To use ES lines to make conventional KO mice, the current homologous recombination system utilizes positive selection with Neo cassette. Sometimes this Neo gene effects other gene expressions nearby but deletion of this gene will need additional mating with Cre-expressing mice, which takes extra 2-3months. TALEN system does not need this step.

Although gene targeting of mice by TALENs has been reported as an alternative of ES method, application of TALENs to mice genetic studies should provide us a huge advantage to overcome difficulties on generating KO mice as follows.

miRNAs are often located in the intron region of host genes. Current technique using ES cells and recombination will leave lox genes which may affect host gene expression level. In this regard, application of TALEN for targeting miRNAs that provides to very short deletion in genome without any additional sequences should be very useful without hurting backbone gene expression.

Furthermore, in general, inter gene regions, including enhancer sequences, and some specific regions of genes such as genes in Y chromosome may contain relatively high repeat sequences, which sometimes leads very low efficiency of homologous recombination. TALEN system is targeting very specific but short genome sequences, thus, this problem can also be solved.

TALEN application will be useful in other aspects of genetic researches using mice. It will be also applicable to make domain specific dominant negative (or active) deletion mice, when in-frame mutation is introduced in the specific region of gene. In addition, to make double knockout mice of mutually closely located two genes used to be a painful work, as cross of each KO mice cannot generate double KO (DKO) and thus need to establish new ES lines from one of KO mice. Application of TALEN system may overcome this problem.

TALEN method will provide us the chances to expand our genetic research using mice in a high throughput way.
